# On Flushing

**Published:** 1891-11-07

**Authors:** 


### ON FLUSHING.

A very little experience of out-patient work in any hospital s sufficient to show what a large proportion of women suffer from flushing. Thus a patient may complain of sudden flushes of heat coming over her, followed by sweating, and this by a sensation of cold during which she shivers.

These phenomena have recently been thoroughly investigated by Dr. H. Campbell, who has published his researches in a most interesting work,* from which we take the following remarks :—

A "flush" is to be distinguished from an ordinary "blush," which is always excited by an emotion, whilst the former is usually independent of any exciting emotional cause. As in the case of a blush there happens a series of cerebral changes, and those occurring in, or referred to the skin, are by no means the sole, but merely the more obtrusive results. It may be well, however, on this account, to take them first. There are commonly, as in the above described case, three successive "skin phenomena" (as for shortness sake they may be called)—heat, sweat, cold, and usually in this order. There may be, however, only two phases, heat-sweat or heat-cold ("dry flush"), or sweat-cold (so called "cold sweat"), or more rarely, only one of the above phases.

The transition between the phases is gradual, thus the feeling of cold may begin in some part of the body before that of heat has quite passed away from other parts.

The relation of the sweating to the hot and cold stages varies to a great degree. It may begin as soon as the sensation of heat, or later on, sometimes only as the cold feeling comes on.

The "heat" may affect instantaneously all the area of skin it implicates; more commonly, however, it starts in some region of the skin and spreads in a more or less definite direction. As to the directions of spreading most interesting facts have been elicited. It may be a "descending flush," in which case it always begins in the head and spreads down the body, stopping short at some or other level; or, on the other hand, it may be an "ascending flush," when it starts in one or other region of the skin, most commonly the chest, abdomen, or back, and ascends—and in this case it always ascends to the face and head. These phenomena are very analogous to sensory epileptic auræ, which may spread in precisely the same way—apparently owing to the cerebral representation of sensory nerves of the skins being as continuous as their cutaneous distribution. Why a flush, which begins below, always ascends to the head and face, is not so certain—possibly, as Campbell remarks, it may be due to the greater instability of the sensory centres for the upper part of the body, insomuch as these have become more closely associated with the expression of mental states, especially with those which have a well marked emotional character. Various names are given by patients to the sensation of heat experienced—*e.g.*, it may be so severe as to be termed "scalding," "burning"—whatever its intensity, it is always felt most in certain parts—the face, neck, and hands.

Though there is this feeling of heat of the skin, there is by no means always dilatation of the skin blood vessels—the whole area of skin implicated may be quite pale. And, as a rule the vaso-motor dilatation is confined to the exposed parts—face, neck, and sometimes hands. The sudden flushing with hot blood of these areas of skin will cause afferent impulses to pass up to the brain, and so increase the feeling of heat which, primarily, occurs quite independently of the state of the skin. The above-mentioned reason is probably also accountable for the greater intensity of the sensation of heat, and for the vaso-motor dilatation in the face, neck, and in a less degree, hands. The sweating which, as mentioned above, may begin at any period in the hot stage, may be of very varying intensity—its amount and area over which it occurs has no relation to the intensity of the sensation of heat, nor to the area in which this is felt—thus a slight flush may be followed by most copious perspiration, which may even be so profuse as to make a change of clothes necessary.

The sweating is always most at night, probably owing to the skin being then warmer than at other times.

The sensation of cold, which succeeds that of heat, does not, as a rule, spread in any definite direction, but generally affects the whole body, most felt, however, in certain parts, especially the back. It may be so intense as to cause the patient to shiver. The skin during this stage may be quite normal, but generally there is vaso-motor constriction of the cutaneous vessels.

But phenomena other than those referable to the skin occur; thus, a feeling of faintness or sudden weakness is almost invariable during the hot stage. The person may catch hold of some support, or drop anything she holds, but rarely falls. This feeling of weakness, too, often causes tremor of the muscles, more particularly those of the knees, and the whole body may tremble.

Again, a feeling of suffocation, with a great desire for fresh air, is a most common accompaniment of a flush, and may cause the patient to rush to open the window. Or it may take the form of a feeling of being stifled or choked, very much as an hysterical attack. Palpitation is often complained of; so also is nausea, though actual vomiting is rare. In many cases, however, the nausea complained of is merely a symptom of the dyspepsia which may cause the flushes, and not a part of the actual nerve-storm. Similarly, also, as regards pain, for, probably, any severe pain may, in a predisposed subject, set up a flush, though in some cases it may be part of the flush. Sudden dimness of vision and a feeling of giddiness are also sometimes marked features.

In addition to these sensory and motor features of a flush, intellectual and emotional changes may occur; for instance, a feeling of lowness of spirits.

With regard to all these phenomena, it is first to be remarked that they generally occur during the hot stage, though not invariably, for they may be experienced after or before this and also in nerve-storms, where the feeling of heat and dilatation of the cutaneous blood vessels is very little marked or absent.

Hence, it cannot be held that, *e.g.*, the feeling of faintness is due to anæmia of the brain, the result of dilatation of the blood vessels of the skin. The storm is not merely a vaso-motor one, it is a nerve-storm affecting most varied cortical centres, among which the vaso-motor ones are generally, though not invariably, included, much more commonly, indeed, than in the case of the more or less allied nerve-storms of megrim, epilepsy, or hysteria.

The causes of these peculiar and most troublesome nerve-storms are most varied. They are divided by Campbell into predisposing—under which he includes sex, age, menstruation and its irregularities, climacteric, pregnancy, and lactation—and exciting, under which he includes emotion, indigestion, and heat.

Owing to the length of this paper these can only be very shortly touched on here. The female sex is very much more liable to flushes than the male, and men who suffer from them are often of a nervous, emotional temperament. The age at which the flushes occur is from a few years before puberty to many years past the climacteric, but the most liable period is the climacteric, few women escaping them then. The irritability of the central nervous system, of which these flushes are one of the results, begins some years before the climacteric, at 35 or 36, and gradually increases up to that period, after which it gradually declines. Flushes, again, have a close connection with menstruation; they are very apt to occur during the menstrual period, and especi-

* "Flushing and Morbid Blushing," by Dr. H. Campbell.

ally if there be menstrual irregularities. These may in some cases be the direct causes of the state of the central nervous system, of which flushings are one expression; but the reverse is much more often true, *i.e.*, in women there is, normally, a monthly cycle of changes in the nervous system, of which menstruation is only one of the results; and at certain periods in this rhythm the nervous system is in such a state, that flushes are apt to occur; whilst if the rhythm be disturbed, irregular menstruation and flushes are some of the expressions of this. This nervous rhythm goes on for many years (often until the end of life), after actual menstruation has ceased; thus flushes are peculiarly liable to occur at what would have been menstrual periods after the climacteric.

Treatment, regarding which space does not permit us to say anything, may be directed towards the flushes, though with but small results; but it must rather deal with the above mentioned causes of the underlying state of the central nervous system when they are such as to be amenable to treatment.

Of exciting causes none is more frequent than dyspepsia; heat, too, is often an exciting cause. It is interesting, in connection with this latter cause, to note how peculiarly sensitive to heat, and insensitive to cold, many women are during the years preceding and succeeding the climacteric.

